# Screening of cytotoxic or cytostatic flavonoids with quantitative Fluorescent Ubiquitination-based Cell Cycle Indicator-based cell cycle assay

**DOI:** 10.1098/rsos.181303

**Published:** 2018-12-19

**Authors:** Young-Hyun Go, Hyo-Ju Lee, Hyeon-Joon Kong, Ho-Chang Jeong, Dong Young Lee, Soon-Ki Hong, Sang Hyun Sung, Ok-Seon Kwon, Hyuk-Jin Cha

**Affiliations:** 1 College of Natural Sciences, Department of Life Sciences, Sogang University, Seoul, Republic of Korea; 2 College of Pharmacy and Research Institute of Pharmaceutical Sciences, Seoul National University, Seoul, Republic of Korea

**Keywords:** cell cycle, flavonoid, FUCCI, screening, SAR, G2 arrest

## Abstract

The Fluorescent Ubiquitination-based Cell Cycle Indicator (FUCCI) system can be used not only to study gene expression at a specific cell cycle stage, but also to monitor cell cycle transitions in real time. In this study, we used a single clone of FUCCI-expressing HeLa cells (FUCCI-HeLa cells) and monitored the cell cycle in individual live cells over time by determining the ratios between red fluorescence (RF) of RFP-Cdt1 and green fluorescence (GF) of GFP-Geminin. Cytotoxic and cytostatic compounds, the latter of which induced G2 or mitotic arrest, were identified based on periodic cycling of the RF/GF and GF/RF ratios in FUCCI-HeLa cells treated with anti-cancer drugs. With this cell cycle monitoring system, ten flavonoids were screened. Of these, apigenin and luteolin, which have a flavone backbone, were cytotoxic, whereas kaempferol, which has a flavonol backbone, was cytostatic and induced G2 arrest. In summary, we developed a system to quantitatively monitor the cell cycle in real time. This system can be used to identify novel compounds that modulate the cell cycle and to investigate structure–activity relationships.

## 1. Introduction

Natural compounds are historically important resources to identify novel lead structures for the development of anti-cancer therapeutics. More than 60% of currently used anti-cancer drugs originate from natural sources [1]. A library of natural compounds with unique chemical structures has been established. However, to develop novel anti-cancer drugs, these phytochemicals are usually screened using cancer cell death as a readout [2,3]. Taxol, one of the most successful FDA-approved anti-cancer drugs, was identified via this method [4]. Development of a high-throughput screening platform that uses a different readout (other than cell death) will help to identify novel therapeutic phytochemicals.

Flavonoids, which have a phenylchromanone structure (C6-C3-C6) and at least one hydroxyl group, are among the most abundant phytochemicals in vegetables and fruits. Anthoxanthins (also called bioflavonoids) are categorized into flavones (2-phenylchromen-4-one: apigenin (API), chrysin, and luteolin (LUT)) and flavonols (3-hydroxyl-2-phenylchromen-4-one: kaempferol (KP), quercetin (QC), and myricetin (MC)), the latter of which has a 3-hydroxyl group in the central pyran-4-one ring. The anti-cancer activity of bioflavonoids has been extensively studied [3] and is due to the induction of cell death [5] or cell cycle arrest at G1/S or G2/M phase [6]. Flavonoids elicit different effects on cancer cells according to their structures [7,8]. Binding of flavonoids to β-amyloid [9] and their antioxidative capacity are closely associated with the number of hydroxyl groups [10]. Therefore, the chemical structure of a flavonoid may determine its biological effect toward cancer cells. This notion is supported by the finding that QC, but not KP, is cytotoxic to human pluripotent stem cells (hPSCs) [11], which implies that the hydroxyl groups in a flavonoid are an important determinant of its biological activity. However, the correlation between the structures of flavonoids and their anti-cancer effects remains to be determined.

Flavopiridol (also known as alvocidib), a flavonoid derived from an indigenous plant, was identified as a potent inhibitor of cyclin-dependent kinases [12] and was approved for the treatment of acute myeloid leukaemia by the FDA [13]. Phytochemicals have been extensively screened to identify novel cell cycle modulators that can be used to develop chemotherapeutics [14]. However, conventional assays to monitor each stage of the cell cycle, such as propidium iodide-based fluorescence-activated cell sorting and immunoblotting analysis of specific marker proteins, are not appropriate to screen a large number of compounds.

In this study, we used the Fluorescent Ubiquitination-based Cell Cycle Indicator (FUCCI) system to monitor cell cycle progression in live cells [15] and determined periodic changes in the cell cycle based on the ratios between red fluorescence (RF) of Cdt1 and green fluorescence (GF) of Geminin in synchronized FUCCI-expressing HeLa cells (FUCCI-HeLa cells). Using this system, we screened 10 in-house flavonoids to identify cell cycle inhibitors. API and LUT, which have a flavone backbone, elicited cytotoxic effects on various cancer cell lines, whereas KP which has a flavonol backbone, elicited cytostatic effects. Thus, the small molecules that interfere with the cell cycle, were readily identified by simply determining the ratios between RF and GF. This approach will be useful for high-throughput screening of chemical libraries.

## 2. Material and method

### 2.1. Cell culture and reagents

FUCCI-HeLa cells (kindly provided by Dr SG Hwang (Korea Institute of Radiological and Medical Sciences, Seoul, Rep. of Korea)) [16] were maintained in high-glucose DMEM (Gibco, #11995) with 10% fetal bovine serum and 0.1% gentamycin (Gibco, #15750-060) at 37°C in a humidified 5% CO_2_ incubator. Kaempferol (#11852) was purchased from Cayman Chemical Company. Luteolin (#L9283), apigenin (#10798), quercetin (#Q4951), chrysin (#C80105), propidium iodine (#P4170), nocodazole (#M1404), etoposide (#E1383) and camptothecin (#C9911) were purchased from Sigma-Aldrich. MLN8237 (#S1133) was purchased from Selleck Chemicals. Thymidine (#194754) was purchased from MP Biomedicals. Hoechst 33342 (#H21492) was purchased from Invitrogen.

### 2.2. Immunoblotting and immunofluorescence

Antibodies for Securin (#sc-56207), Cyclin B1 (#sc-245) and β-actin (#sc-47778), were purchased from Santa Cruz Biotechnology. Antibodies for TPX2 (#12245S), phospho-Histone H3 (#14955) and cleaved caspase-3 (#9664S) were purchased from Cell Signaling Biotechnology. Immunoblotting and immunofluorescence assay were performed as described previously [17].

### 2.3. Cell synchronization

FUCCI-HeLa cells were synchronized at the G1/S boundary by using double thymidine block (DTB) as described previously [18]. In detail, cells were treated with 2.5 mM thymidine for 16 h and were released back with normal medium for 8 h. After second thymidine treatment with 2.5 mM thymidine for an additional 16 h, cells were arrested at G1/S boundary. Cells were live monitored with the JULI-stage live image machine (NanoEntek).

### 2.4. Time-lapse imaging and compensation

Time-lapse images were acquired at constant time points (Bright field (BF), GFP, and RFP 3-channel. LED power: 1, Bright: 4, RFP is 7. Exposure: 400 ms, BF is 70 ms. Images captured with auto-focusing in all channels and cycles.) using JULI-stage software (NanoEntek), and contrast of both green and red fluorescence compensated using batch editor of photoscape software (Auto contrast: middle. Contrast: low, +5. Erode). Attached cell counter of JULI-stat (NanoEntek) was used as analysing software (intensity of min: 10, max: 255, counted for each pixel).

### 2.5. Cell proliferation quantification

For all of the cell growth analysis, JULI-stage and JULI-stat software were used for live imaging and cell quantification according to the manufacture's protocol.

### 2.6. Flow cytometry

Cells were initially synchronized with double thymidine block, and then collected at respective time points after the release from this block. The cells were washed in PBS twice, and then fixed with chilled 70% ethanol at 4°C overnight. The fixed cells were rinsed in PBS twice, and then incubated with RNaseA (10 µg ml^−1^, Invitrogen, 12091-021) and propidium iodide (50 µg ml^−1^, Sigma, P4170) at 37°C for 30 min in dark room, followed by analysis on a BD FACSCalibur (BD Biosciences).

### 2.7. Annexin V staining

For Annexin V staining, cells were washed twice with PBS and were stained with FITC-Annexin V and 7-AAD for 30 min at RT in dark. Cell death was analysed with FACS according to apoptosis detection kit I protocol (BD Bioscience).

### 2.8. Statistical analysis

The graphical data were presented as mean ± s.e.m. Statistical significance among the three groups and between groups was determined using one-way analysis of variance (ANOVA) following Bonferroni post-test and Student's *t*-test respectively. Significance was assumed for *p* < 0.05 (*), *p* < 0.01 (**), *p* < 0.001 (***).

## 3. Results

### 3.1. Determination of the cell cycle stage of FUCCI-HeLa cells based on the RF/GF and GF/RF ratios

**Figure 1.**
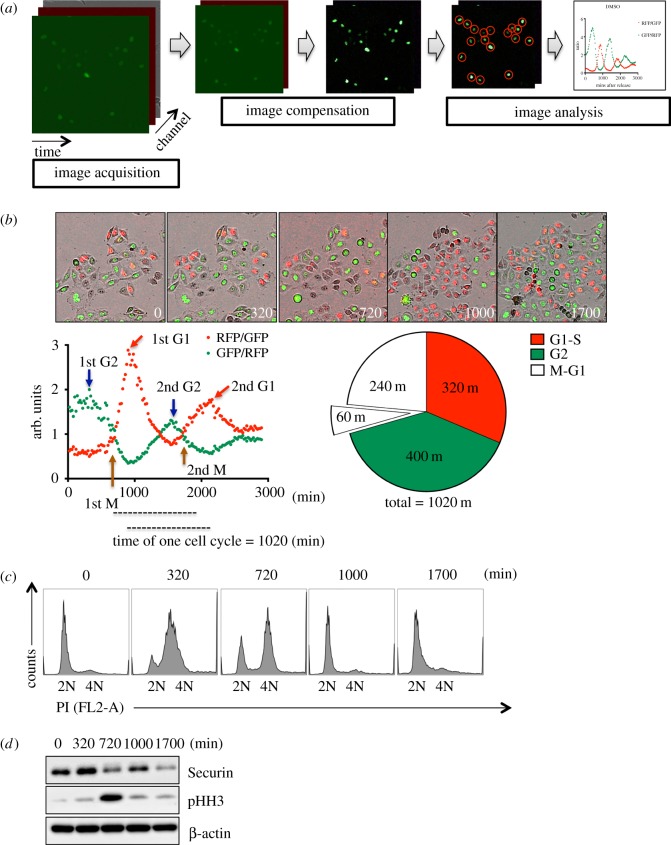
Determination of the cell cycle stage of FUCCI-HeLa cells based on the RF/GF and GF/RF ratios. (*a*) Schematic procedure of measuring RF/GF ratio in FUCCI-HeLa. In short, the process includes the live-image acquisition, image compensation and analysis. Detail procedure is described in Materials and methods section. (*b*) Florescent images of FUCCI-HeLa at indicative time of release from G1/S arrest (minutes) (top). Graphical presentation of time dependent RF/GF and GF/RF ratio of each cell image after DTB release (bottom left). The circular chart represents the approximate length of one cell cycle stages of FUCCI-HeLa (bottom right). (*c*) Flow cytometry of time dependent cell cycle profiling of control HeLa after DTB at indicative time (minutes) (2N for G0/G1 and 4N for G2/M). (*d*) Immunoblotting analysis for Securin and phospho-Histone H3 (pHH3) in synchronized HeLa after DTB release at indicative time (minutes) after G1/S release, β-actin for equal protein loading control.

The intensities of GF of Geminin and RF of Cdt1 in FUCCI-HeLa cells fluctuate according to the cell cycle stage [15]. Thus, the cell cycle stage of each cell can be predicted by simply measuring the intensities of GF and RF. A single clone of FUCCI-HeLa cells (clone #8) was isolated (electronic supplementary material, figure S1A and movie S1). Fluorescence images of this clone (hereafter referred to as FUCCI-HeLa cells) were obtained in real time and processed (figure 1*a*). Cells were synchronized via double thymidine block (DTB) to induce arrest at the G1/S boundary, and the average intensities of GF and RF in FUCCI-HeLa cells at each cell cycle stage were determined (electronic supplementary material, figure S1B and movie S2). When the ratio of the RF intensity versus the GF intensity (RF/GF) and the ratio of the GF intensity versus the RF intensity (GF/RF) were plotted over time after G1/S release, these values fluctuated according to the cell cycle stage. The GF/RF and RF/GF ratios were highest in G2 and G1 phases, respectively, of the first cell cycle (approximately 17 h) after G1/S release (figure 1*b*). This fluctuation of the GF/RF and RF/GF ratios was significantly attenuated in the second cell cycle (figure 1*b*) and was not observed in subsequent cell cycles, implying that cells became asynchronized after the first cell cycle following DTB release.

To confirm that the GF/RF and RF/GF ratios accurately predict the cell cycle stage, the cell cycle status of HeLa cells, whose cell cycle profile was identical to that of FUCCI-HeLa cells (electronic supplementary material, figure S1C), was determined after DTB release. As expected, the timings of the first G2 and G1 phases correlated with the timings when the GF/RF and RF/GF ratios peaked, respectively (figure 1*c*). At the time point when the GF/RF and RF/GF ratios were similar after the first G2 phase (approximately 720 min after DTB release), most cells were in mitosis (figure 1*b*), as revealed by the high level of phospho-Histone H3 (pHH3), a typical mitotic indicator (figure 1*d*).

### 3.2. Effects of small molecules on the cell cycle

**Figure 2.**
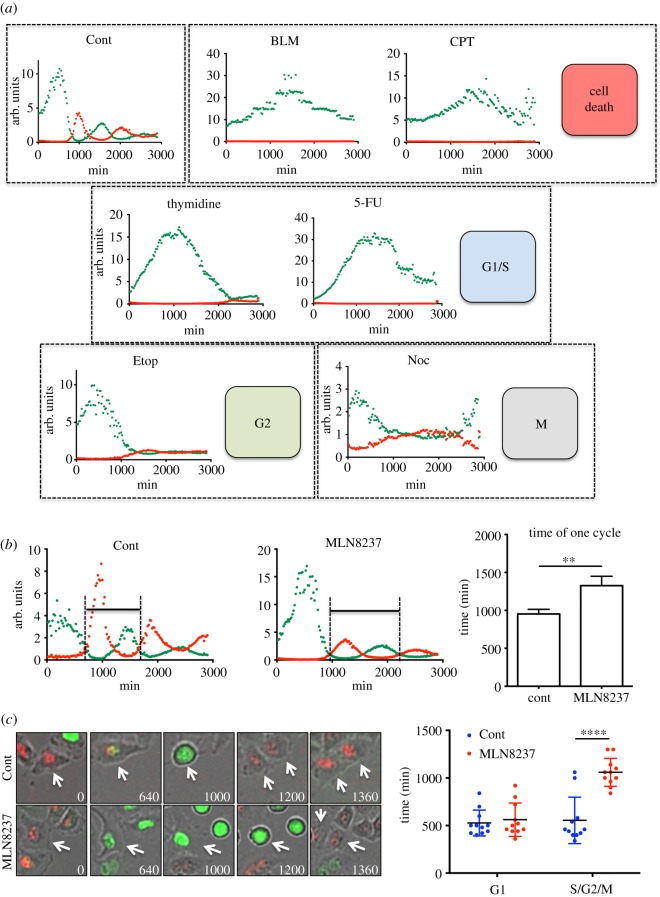
Effects of small molecules on the cell cycle. (*a*,*b*) Profiles of RF/GF ratio of FUCCI-HeLa after treatment of Bleomycin (BLM: 5 µg ml^−1^) or Camptothecin (CPT: 500 nM), Thymidine (2.5 mM), 5-Fluorouracil (5-FU: 1 µM), Etoposide (Etop: 500 nM), Nocodazole (Noc: 50 ng ml^−1^) (*a*) and MLN8237 (200 nM) (*b*) after release from G1/S compared to the control are shown. Time frame for one cell cycle in RF/GF ratio profile (black bar, left). Average time frame of one cell cycle of control (Cont) or MLN8237 (200 nM) treatment is graphically presented (right). (*c*) Representative fluorescent images from DMSO control (Cont) or 200 nM of MLN8237 treatment (MLN8237) at indicated time after G1/S release (minutes); white arrows indicate the representative cell, measured for time frame of cell cycle (left). Graphical presentation of time frame of G1 phase or S/G2/M phase of control (Cont) and MLN8237 treatment (MLN8237) (*N* = 11) (right).

Analysis of the RF/GF and GF/RF ratios revealed the cell cycle stage of FUCCI-HeLa cells (figure 1). Therefore, we next investigated whether this approach could be used to predict the effects of chemical compounds on the cell cycle. To this end, the RF/GF and GF/RF ratios were determined in FUCCI-HeLa cells treated with various small molecules affecting cell cycle such as thymidine (for G1/S), 5-FU (for G1/S), nocodazole (for M phase) and the genotoxic reagents etoposide (Etop for G2) and bleomycin (BLM), and camptothecin (CPT). While treatment with BLM and CPT also changed the fluorescence ratios by inducing cell death following G2 arrest, other treatments revealed typical G1/S, G2 and M phase arrest profile (figure 2*a*).

Next, we investigated whether this approach can be used to monitor mitotic delay following perturbation of mitotic kinases such as Aurora-A kinase, a critical player in mitotic entry [19]. As expected, treatment with MNL8237, an Aurora-A kinase inhibitor, delayed the timing of the peak in mitotic cells (approx. 700 min for control cells versus approx. 1000 min for MNL8237-treated cells) and increased the duration of one cell cycle (approx. 1000 min for control cells versus approx. 1300 min for MNL8237-treated cells) (figure 2*b*). MNL8237 treatment significantly increased the duration from S phase to M phase, but did not affect that from G1 phase to S phase (figure 2*c*).

### 3.3. Screening of an in-house flavonoid library

**Figure 3.**
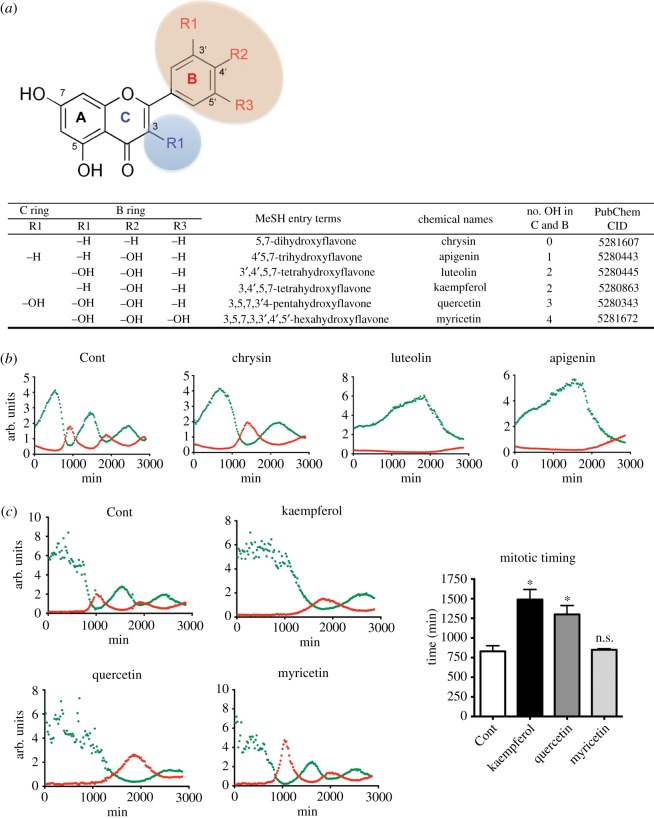

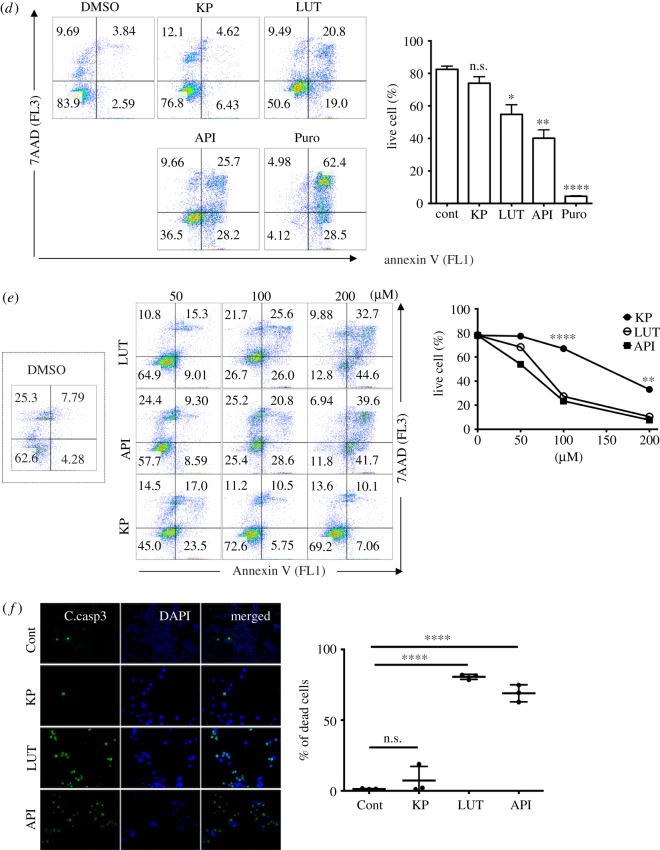
Screening of an in-house flavonoid library. (*a*) Chemical structure (top) and table (bottom) for flavonoids used in this study. (*b*,*c*) Profile of RF/GF ratio of FUCCI-HeLa under treatment of 50 µM of flavone (chrysin, luteolin and apigenin: *b*) or flavonol (kaempferol, quercetin and Myricetin: *c*) after release from G1/S compared to the DMSO control. Mitotic timing (time point when mitotic population is enriched) after treatment of flavonols is graphically presented (*c*: right, ns: not significant). (*d*,*e*) Flow cytometry for Annexin V and 7AAD after treatment of 50 µM of kaempferol (KP), luteolin (LUT), and apigenin (API) in HeLa (left). Double negative population (Live cells) to Annexin V and 7AAD staining is graphically presented (right). Puromycin (Puro: 5 µg ml^−1^) was used for positive control of cell death. (*e*) Live cell population (double negative to Annexin V and 7AAD) after treatment of indicated dose of flavonoids (left) is graphically presented (right). (*f*) Fluorescent microscopic images of cleaved caspase 3 (C.casp3: Green) positive population after treatment of 50 µM of kaempferol (KP), luteolin (LUT), and apigenin (API) (left). Percentage of cleaved caspase 3 positive cells (e.g. dead cells) is graphically presented (ns: not significant, right).

The effects of chemical compounds on the cell cycle were determined by measuring the fluorescence ratios (figure 2). Therefore, we next sought to identify a flavonoid(s) that affects the cell cycle from an in-house library. Ten flavonoids (electronic supplementary material, figure S2A) with different numbers of hydroxyl groups (figure 3*a*) were screened. According to alteration of profile of florescence ratio after treatment of API, LUT or Chrysin, which have a flavone backbone, treatment of API or LUT may cause cell death, of which profile was similar to that of BLM and CPT as shown in figure 2*a*, while Chrysin treatment delays mitotic progression (figure 3*b*). The cell cycle profile was affected less by treatment with KP, QC and MC, which have a flavonol backbone. Treatment with KP and QC significantly delayed mitotic entry, whereas treatment with MC, which has one more hydroxyl group, only marginally affected the cell cycle (figure 3*c*). The effect of flavonoids on FUCCI-HeLa cells was probably dependent on the number of hydroxyl groups in the B and C rings (figure 3*a*) because treatment with MC, which has four hydroxyl groups in total, did not affect mitotic timing (figure 3*c*).

The fluorescence ratios were dramatically changed upon cell death (figure 2*a*). Therefore, we next examined the effect of API and LUT on cell death, rather than on cell cycle arrest. As expected, treatment with API and LUT, but not KP, killed cells, as determined by Annexin V/7AAD staining (figure 3*d*,*e*), cleaved caspase 3 staining (figure 3*f*), and observation of cellular morphology (electronic supplementary material, figure S2B). We speculate that flavonoids with a flavonol backbone (KP, QC and MC) are not cytotoxic, unlike flavonoids with a flavone backbone (API and LUT), despite the fact that API and KP both have one hydroxyl group in their B ring, whereas LUT and QC both have two (electronic supplementary material, figure S2C). On the other hand, flavonoid glycosides, which are common components in many plants, only marginally affected or did not affect the cell cycle, regardless of the number of hydroxyl groups (electronic supplementary material, figure S2D).

### 3.4. Induction of G2 arrest by KP

**Figure 4.**
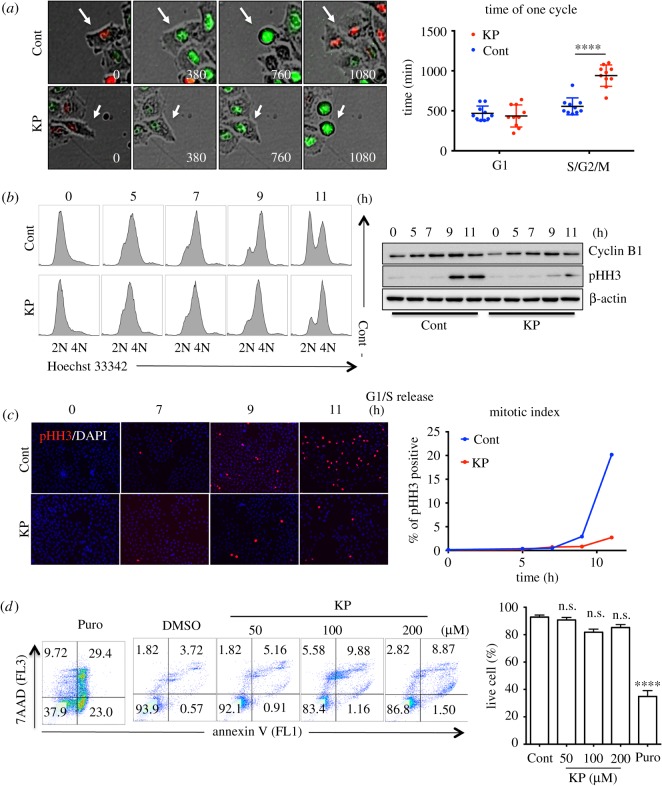
Induction of G2 arrest by KP. (*a*) Time-lapse fluorescent microscopic images of FUCCI-HeLa after G1/S release after treatment of DMSO (Cont) or kaempferol (KP) (left). Duration of G1 phase (G1) and S to M phase (S/G2/M) is graphically presented (right) (*N* = 10). (*b*) Flow cytometry of time dependent cell cycle profiling of control HeLa at indicative time (hours: h) after G1/S release with DMSO (Cont) or kaempferol (KP: 50 µM) treatment (left). Immunoblotting analysis for Cyclin B1 and pHH3 of HeLa at indicative times (h) after G1/S release, β-actin for equal protein loading control. (*c*) Cells in the mitosis, positively stained with pHH3 antibody (red) were counted at indicative time (h) after G1/S release with DMSO (Cont) or kaempferol (KP: 50 µM) treatment (left). Mitotic index is graphically presented (right). (*d*) Flow cytometry for Annexin V and 7AAD after treatment of indicative dose of kaempferol (KP) (left). Double negative population (live cells) to Annexin V and 7AAD staining is graphically presented (right, ns: not significant). Puromycin (Puro: 5 µg ml^−1^) was used for positive control of cell death.

According to the fluorescence ratios after treatment with flavonols (KP, QC and MC) (figure 3*c*), the timing of the peak in mitotic cells, which was normally approximately 720 min after G1/S release (figure 1*b*), was markedly delayed by KP treatment (figure 3*c*). This result suggests that KP induces G2 arrest. To confirm this, cell cycle transition was monitored in live FUCCI-HeLa cells treated with KP. KP-treated FUCCI-HeLa cells remained in G2 phase, as demonstrated by the increase in the GF intensity, while control cells progressed into mitosis as normal (figure 4*a*). Measurement of the duration from S phase to M phase, during which GF was detected, demonstrated that KP inhibited G2-to-M transition (figure 4*a*). This result was confirmed by conventional approaches to detect G2 arrest, such as propidium iodide-based fluorescence-activated cell sorting, immunoblotting for mitotic marker proteins (e.g. Cyclin B1 and pHH3) (figure 4*b*), and determination of the mitotic index via pHH3 staining (figure 4*c*). Treatment with relatively high concentrations of KP (up to 200 µM) only slightly increased cell death (figure 4*d*), suggesting that this compound mainly inhibits cancer cell growth by inducing G2 arrest (figure 3*e*).

### 3.5. Inhibition of tumour growth by flavonoids

**Figure 5.**
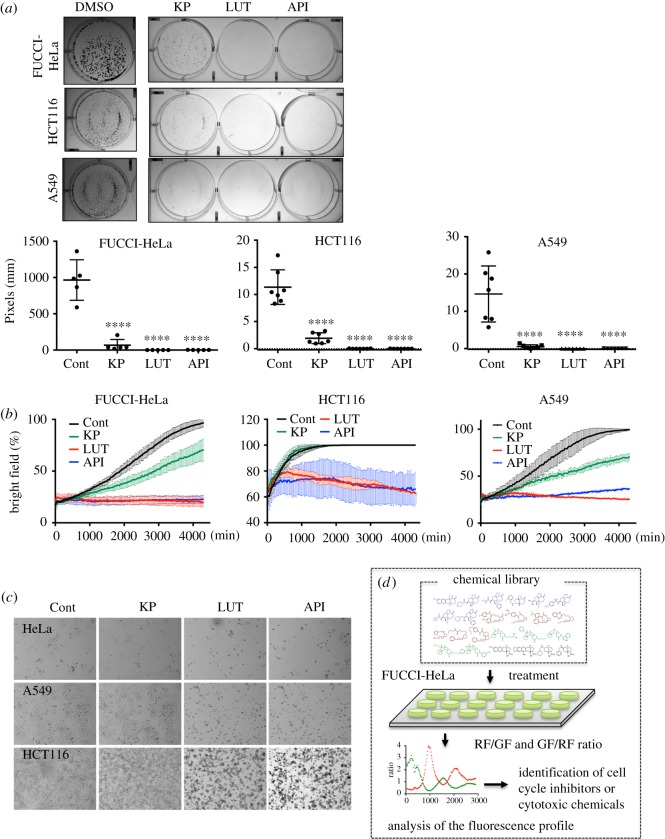
Inhibition of tumour growth by flavonoids. (*a*) Representative images of clonogenic assay (top) and quantification data (bottom) of FUCCI-HeLa, HCT116 and A549 cancer cells at 7 days after treatment of 50 µM of kaempferol (KP), luteolin (LUT) and apigenin (API). (*b*) Time dependent growth curve of indicative cancer cell lines under treatment of 50 µM of kaempferol (KP), luteolin (LUT) and apigenin (API). (*c*) Microscopic images of HeLa, A549 and HCT116 cancer cells at 72 h after treatment of 50 µM of kaempferol (KP), luteolin (LUT) and apigenin (API). (*d*) Graphical scheme of FUCCI-HeLa-based system for screening the small molecules for identification of novel cell cycle inhibitor.

Flavonoids with anti-cancer effects identified based on the cell cycle profile of FUCCI-HeLa cells were also effective against HCT116 and A549 cancer cells. According to clonogenic (figure 5*a*) and proliferation (figure 5*b*) assays, flavonoids (KP, LUT and API) significantly attenuated cancer cell proliferation, although the inhibitory effects of LUT and API, which have a flavone backbone, were greater than that of KP, which has a flavonol backbone. Moreover, LUT and API were cytotoxic to these cancer cells (figure 5*c*), consistent with previous data (figure 3*d*,*e*). Taken together, these results demonstrate that screening based on the cell cycle profile of FUCCI-HeLa cells, as judged by periodic changes in the intensities of GF and RF, is a useful and efficient means to identify novel small molecules that elicit cytotoxic or cytostatic effects on cancer cells (figure 5*d*).

## 4. Discussion

FUCCI system has been extensively used in not only of cell biology of cell cycle in stem cells [20,21] and cancer [16], but also theoretical modelling of cell cycle [22] and cell migration [23]. Moreover, efficacy of cancer therapeutics [24], cancer mobility [25] or even angiogenesis [26] in three-dimensional culture, was examined using FUCCI system [27,28], which may contribute to anti-cancer drug development. We used the FUCCI system to monitor the cell cycle in real time and thereby rapidly identify cell cycle modulators from an in-house library of flavonoids. To examine the cell cycle, we measured GF and RF in synchronized FUCCI-HeLa cells in real time, plotted the RF/GF and GF/RF ratios over time, and determined the timings of the first G2 and M phases and the second G1, G2 and M phases, as well as the duration of one cell cycle (figure 1). Typical profiles of the fluorescence ratios upon G2 or M arrest or cell death were obtained by treating FUCCI-HeLa cells with various chemotherapeutics (figure 2). These results were used to predict whether small molecules elicited cytotoxic or cytostatic effects. By screening an in-house flavonoid library using this system, we found that API and LUT, which have a flavone backbone, were cytotoxic, while KP and QC, but not MC, which have a flavonol (or 3-hydroxyflavone) backbone, induced G2 arrest (figure 3). Although flavonoid glycosides did not elicit marked effects regardless of their structures (electronic supplementary material, figure S2A), enteric bacteria hydrolyse these compounds and thereby increase their bioavailability after administration [29]. Thus, the anti-cancer efficacy of flavonoid glycosides should be examined in an animal model but not in cell model, in the future.

The antioxidant activities of flavonoids with different numbers of hydroxyl groups in their B ring [30] and C ring [31] have been investigated. These studies suggest that hydroxyl groups in both rings are critical for antioxidant activity. By contrast, the anti-cancer activities of API and LUT, which are flavones that lack the 3-hydroxyl group in the benzopyrone structure, were greater than those of KP and QC, which are 3-hydroxyflavones or flavonols (figure 5). Similarly, the induction of G2 arrest by flavonols is probably affected by the number of hydroxyl groups in their B ring because treatment with MC, which has three hydroxyl groups in its B ring, only marginally affected the cell cycle (figure 3*c*), whereas several studies demonstrated that this compound has anti-cancer activity [32]. MC induces death of pancreatic cancer cells, but not of normal pancreatic ductal cells [33]. We previously demonstrated that QC, which induced G2 arrest in HeLa cells to a lesser extent than KP (figure 3*c*), selectively kills undifferentiated hPSCs, which have the capacity to form teratomas *in vivo*, whereas KP does not affect these cells [11]. Considering the specific effects of flavonoids on tumorigenic cells (cancer cells or hPSCs), the structure–activity relationships of these compounds need to be established in cell types of interest. We studied the effects of flavonoids on the viability of three cancer cell lines, namely, HeLa (a cervical cancer cell line), HCT116 (a colon cancer cell line) and A549 (a lung cancer cell line). These analyses demonstrated that flavonoids with a flavone backbone (LUT and API) and a flavonol backbone (KP) elicited cytotoxic and cytostatic effects, respectively, consistent with the results obtained using the FUCCI system. It is noteworthy that anti-tumour activity of four flavonoids (LUT, API, KP or QC) were validated *in vivo* mouse models [34–37], suggesting the FUCCI-based screening system would be advantageous for the initial ‘Hit’ screening. Considering the recent advance of FUCCI to monitor not only cell migration [38] and angiogenesis [39], but also chemosensitivity in three-dimensional culture system [25], application of FUCCI-based model in drug development would be useful in future.

In summary, profiling of fluorescence ratios in FUCCI-HeLa cells, which enables screening using cell cycle modulation as a readout, will help to determine the relationship between the anti-cancer activity of flavonoids and their structures and to identify novel cell cycle modulators.

## Supplementary Material

Supplementary figures and figure legends

## Supplementary Material

Supplementary Movie 1

## Supplementary Material

Supplementary Movie 2

## Data accessibility

The raw data for each figure was deposited in Dryad Digital Repository: https://dx.doi.org/10.5061/dryad.40cd5ps [40].
