# Supplementary material for: Screening of cytotoxic or cytostatic flavonoids with quantitative Fluorescent Ubiquitination-based Cell Cycle Indicator-based cell cycle assay
Source: R Soc Open Sci. 2018 Dec 19;5(12):181303. doi: 10.1098/rsos.181303 (PMC6304118; doi:10.1098/rsos.181303)
Supplement: Supplementary figures and figure legends [file rsos181303supp1.pdf]

Supplemental information for

Screening of cytotoxic or cytostatic flavonoids with quantitative FUCCI-based cell cycle assay

Running Title: FUCCI based flavonoid screening

Young-Hyun Go, Hyo-Ju Lee, Hyeon-Joon Kong, Ho-Chang Jeong, Dong Young Lee, Soon-Ki Hong, Sang Hyun Sung, Ok-Seon Kwon and Hyuk-Jin Cha

This PDF file includes:

Supplementary Figure legends

Supplementary Figures 1-2

Supplementary Figure legends

Figure. S1 (A) Schematic of Single cell-derived cloning method. Cultured FUCCI-HeLa cells were trypsinized for single cell plating. A single cell of FUCCI-HeLa cells was plated in each well of 96-wall plate and cultured four weeks. In turn, wells, positive to both GFP and RFP, were selected for further amplification to establish a single cell-derived clone with high intensity of GFP and RFP. Finally, the single clone was established. (B) Fluorescence microscopic images of FUCCI-HeLa, expressing GFP and RFP in each different cell cycle stage after release from DTB. (C) DNA content after staining Hoechst-33342 (2μg/mL) staining of asynchronized HeLa and FUCCI-HeLa single clone was measured by flow cytometry.

Figure. S2 (A) List of flavonoids used in this study (B) Live cell images of FUCCI-HeLa cells after treatment of kaempferol (KP), luteolin (LUT), and apigenin (API) (50μM). (C) Structure and cytotoxicity table of API, KP, LUT, and QC (D) RF/GF ratio of FUCCI-HeLa after treatment of flavone glucoside derivatives.

Movie S1

Time-lapse images of FUCCI-HeLa single clones after double thymidine block (DTB)

Movie S2

Time-lapse images of FUCCI-HeLa single cell

Figure. S1

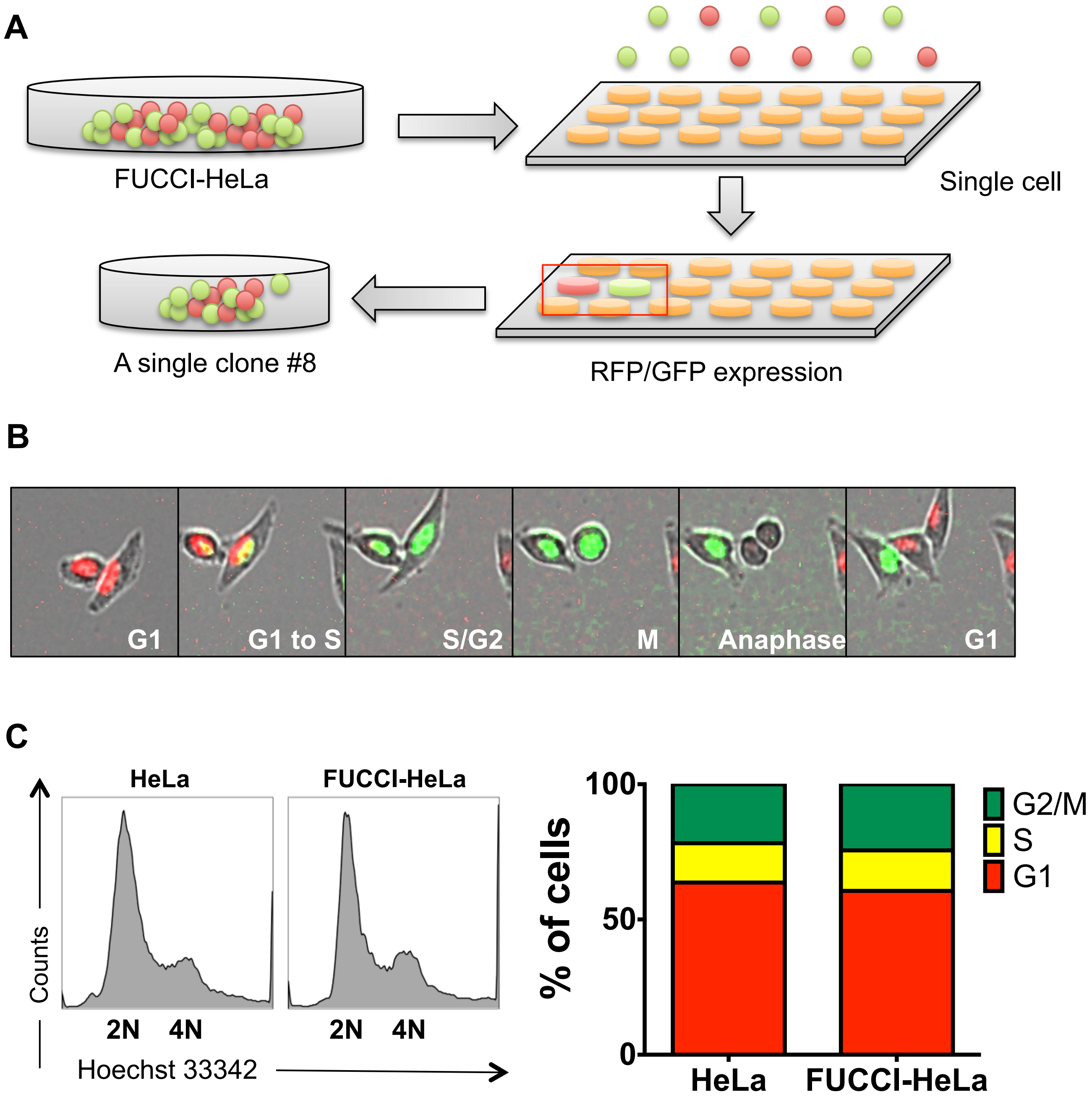
A
FUCCI-HeLa
Single cell
A single clone #8
RFP/GFP expression
B
G1
G1 to S
S/G2
M
Anaphase
G1
C
HeLa
FUCCI-HeLa
Counts
2N
4N
2N
4N
Hoechst 33342
% of cells
100
50
0
HeLa
FUCCI-HeLa
G2/M
S
G1

Figure. S2

A

Code	Name	R_1	R_2	R_3	R_4	R_5	R_6	R_7
F1	Chrysin	H	OH	H	H	H	H	H
F2	Apigenin	H	OH	H	H	OH	H	H
F3	Apigenin 7-*O*-*β*-*D*-glucoside	H	-O-Glc	H	H	OH	H	H
F4	Luteolin	H	OH	H	OH	OH	H	H
F5	Luteolin 7-*O*-*β*-*D*-glucoside	H	-O-Glc	H	OH	OH	H	H
F6	Kaempferol	H	OH	H	H	OH	H	OH
F7	Kaempferol 3-*O*-*β*-D-glucoside	H	OH	H	H	OH	H	-O-Glc
F8	Quercetin	H	OH	H	OH	OH	H	OH
F9	Quercetin-3-*O*-sophoroside	H	OH	H	OH	OH	H	-O-Sophorose
F10	Myricetin	H	OH	H	OH	OH	OH	OH

B

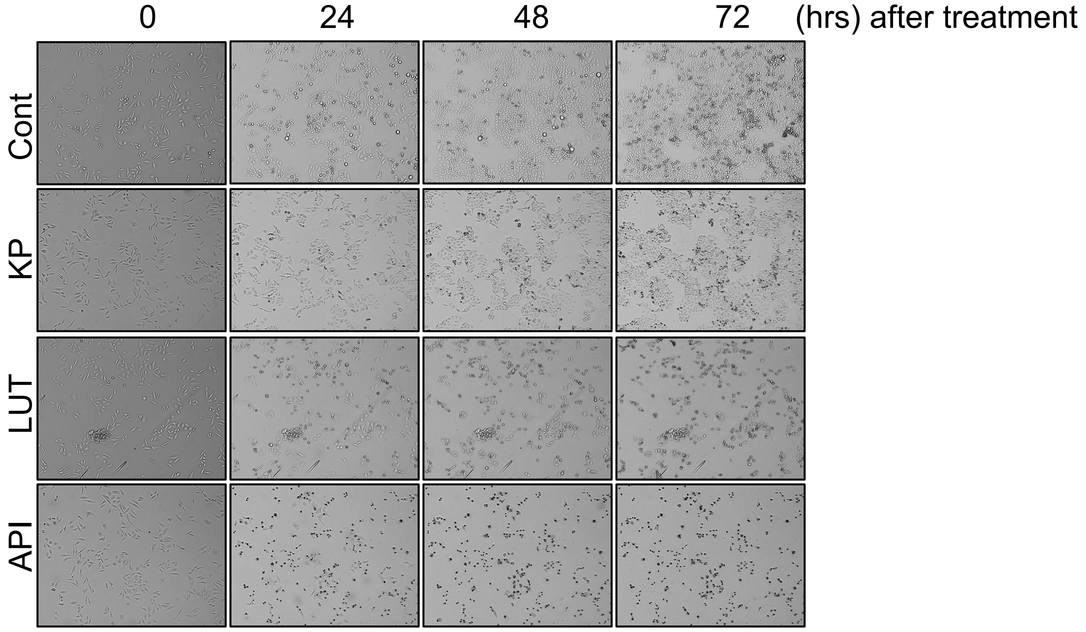

C

B ring			C ring	Chemical Names	Cytotoxicity
**R1**	**R2**	**R3**	**R1**		
-H	-OH	-H	-H	Apigenin (API)	**+++**
			-OH	Kaempferol (KP)	-
-OH	-OH	-H	-H	Luteolin (LUT)	**++**
			-OH	Quercetin (QC)	-

D

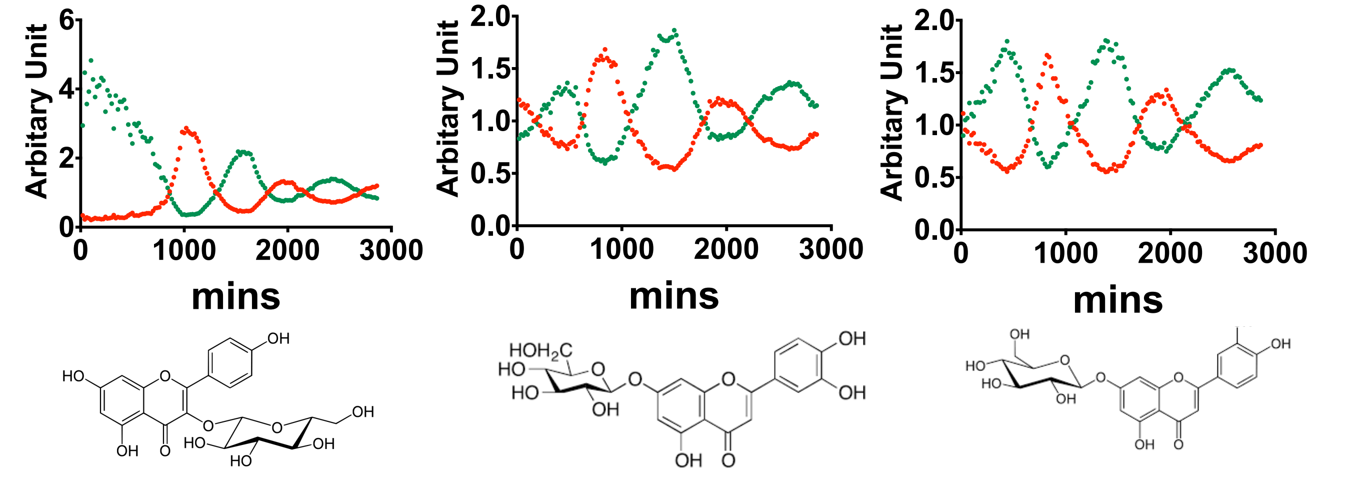

Kaempferol 3-*O*-*β*-D-glucoside   Apigenin 7-*O*-*β*-*D*-glucoside   Luteolin 7-*O*-*β*-*D*-glucoside
